# Physicians' and nurses' opinions on selective decontamination of the digestive tract and selective oropharyngeal decontamination: a survey

**DOI:** 10.1186/cc9180

**Published:** 2010-07-13

**Authors:** Irene P Jongerden, Anne Marie G de Smet, Jan A Kluytmans, Leo F te Velde, Paul J Dennesen, Ronald M Wesselink, Martijn P Bouw, Rob Spanjersberg, Diana Bogaers-Hofman, Nardo J van der Meer, Jaap W de Vries, Karin Kaasjager, Mat van Iterson, Georg H Kluge, Tjip S van der Werf, Hubertus I Harinck, Alexander J Bindels, Peter Pickkers, Marc J Bonten

**Affiliations:** 1 Department of Intensive Care Medicine, University Medical Center Utrecht, P.O. Box 85500, 3508 GA Utrecht, the Netherlands; 2 Department of Perioperative and Emergency Care, University Medical Center Utrecht, P.O. Box 85500, 3508 GA Utrecht, the Netherlands; 3 Laboratory for Microbiology and Infection Control, Amphia Hospital, P.O. Box 90158, 4800 RK Breda, the Netherlands; 4 Department of Intensive Care, Albert Schweitzer Hospital, P.O. Box 444, 3300 AK Dordrecht, the Netherlands; 5 Department of Intensive Care, Medical Center Haaglanden, P.O. Box 432, 2501 CK The Hague, the Netherlands; 6 Department of Anesthesiology and Intensive Care, St. Antonius Hospital, P.O. Box 2500, 3430 EM Nieuwegein, the Netherlands; 7 Department of Intensive Care, Radboud University Nijmegen Medical Center, P.O. Box 9101, 6500 HB Nijmegen, the Netherlands; 8 Departments of Internal Medicine and Pulmonary Diseases and Tuberculosis, University Medical Center Groningen, P.O. Box 30001, 9700 RB Groningen, the Netherlands; 9 Department of Anesthesiology and Intensive Care, Amphia Hospital, P.O. Box 90158, 4800 RK Breda, the Netherlands; 10 Department of Intensive Care, Mesos Medical Center, P.O. box 2500, 3430 EM Nieuwegein, the Netherlands; 11 Department of Intensive Care, Rijnstate Hospital, P.O. Box 9555, 6800 TA Arnhem, the Netherlands; 12 Department of Intensive Care, Diakonessen Hospital, P.O. Box 80250, 3508 TG Utrecht, the Netherlands; 13 Intensive Care Department, Slotervaart Hospital, P.O. Box 90440, 1006 BK Amsterdam, the Netherlands; 14 Department of Intensive Care, Leiden University Medical Center, P.O. Box 9600, 2300 RC Leiden, the Netherlands; 15 Department of Intensive Care, Catharina Hospital, P.O. Box 1350, 5602 ZA Eindhoven, the Netherlands; 16 Department of Medical Microbiology and the Julius Center for Health Sciences and Primary Care, University Medical Center Utrecht, P.O. Box 85500, 3508 GA Utrecht, the Netherlands

## Abstract

**Introduction:**

Use of selective decontamination of the digestive tract (SDD) and selective oropharyngeal decontamination (SOD) in intensive care patients has been controversial for years. Through regular questionnaires we determined expectations concerning SDD (effectiveness) and experience with SDD and SOD (workload and patient friendliness), as perceived by nurses and physicians.

**Methods:**

A survey was embedded in a group-randomized, controlled, cross-over multicenter study in the Netherlands in which, during three 6-month periods, SDD, SOD or standard care was used in random order. At the end of each study period, all nurses and physicians from participating intensive care units received study questionnaires.

**Results:**

In all, 1024 (71%) of 1450 questionnaires were returned by nurses and 253 (82%) of 307 by physicians. Expectations that SDD improved patient outcome increased from 71% and 77% of respondents after the first two study periods to 82% at the end of the study (*P *= 0.004), with comparable trends among nurses and physicians. Nurses considered SDD to impose a higher workload (median 5.0, on a scale from 1 (low) to 10 (high)) than SOD (median 4.0) and standard care (median 2.0). Both SDD and SOD were considered less patient friendly than standard care (medians 4.0, 4.0 and 6.0, respectively). According to physicians, SDD had a higher workload (median 5.5) than SOD (median 5.0), which in turn was higher than standard care (median 2.5). Furthermore, physicians graded patient friendliness of standard care (median 8.0) higher than that of SDD and SOD (both median 6.0).

**Conclusions:**

Although perceived effectiveness of SDD increased as the trial proceeded, both among physicians and nurses, SOD and SDD were, as compared to standard care, considered to increase workload and to reduce patient friendliness. Therefore, education about the importance of oral care and on the effects of SDD and SOD on patient outcomes will be important when implementing these strategies.

**Trial registration:**

ISRCTN35176830.

## Introduction

Respiratory tract infections are a serious threat to patients in ICUs [1,2]. The incidence of these infections can be reduced by use of prophylactic antibiotic regimens, such as selective decontamination of the digestive tract (SDD) [3,4] and selective oropharyngeal decontamination (SOD) [5-7]. The concept of SDD consists of the application of topical (oropharyngeal) and enteral (nasogastric) non-absorbable antimicrobial agents, systemic administration of cephalosporins during the first four days in the ICU and maintaining the anaerobic intestinal flora with a policy favouring antibiotics without anti-anaerobic activity [8]. In SOD, only topical antibiotics in the oropharynx are applied.

The use of SDD and SOD has been the subject of intense controversy, due to methodological issues and concern about increased selection of antibiotic-resistant pathogens [3-5,7-13]. Proponents of the effectiveness of SDD point out beneficial outcomes in individual trials and meta analysis [14], whereas opponents address the lack of sound scientific evidence on patient survival and the constant threat of antimicrobial resistance [15]. Therefore, from May 2004 to July 2006, a large trial was performed in 13 ICUs in the Netherlands in which the effects of SDD and SOD on 28-day mortality were compared with standard care [16]. The trial consisted of three six-month study periods in which either SDD, SOD or standard care was used for all patients in the unit with the order of intervention randomized per centre. SDD and SOD were both effective and associated with a 13% and 11% relative reduction in 28-day mortality, respectively [16].

Bearing in mind the controversy and realizing that both the attitude towards and potential problems with new treatments might seriously affect effectiveness, we determined expectations concerning and experience with SDD as perceived by nursing and medical staff.

## Materials and methods

### Study protocol

Thirteen ICUs participated in the study, differing in size and teaching status and covering all levels of ICU in the Netherlands. Physicians assessed the eligibility of patients for the trial and when eligible confirmed trial medication in the patient chart. Nurses applied oral paste during SDD and SOD and administered suspension and systemic antibiotics during SDD. Furthermore, in all study periods, nurses applied oral hygiene consisting of teeth brushing and cleaning the oral cavity with a dental swab (Table 1).

**Table 1:** Study protocol

Study period	Oral hygiene	Oral paste^†^	Suspension^¥ ^	Cefotaxim*
SDD	+	+	+	+
SOD	+	+		
Standard care	+			

SDD, selective decontamination of the digestive tract; SOD, selective oropharyngeal decontamination.

+ applied four times a day.

^† ^Oral paste consists of polymyxin, tobramycin, amphotericin B and is applied in the oropharynx.

^¥ ^Suspension consists of polymyxin, tobramycin, amphotericin B and is applied in the gastrointestinal tract through a feeding tube.

*Cefotaxim applied intravenous during first four days.

Oral presentations were held at the start of every study period in each of the participating hospitals to inform nursing and medical staff about the trial and the study protocol. Furthermore, posters containing information about the study period were placed visibly in each unit. Both presentations and posters contained non-biased information about the aim of the trial and practical consequences of the next study period (oral hygiene, administration of study medication). Personnel from ICUs that had not used SDD before were invited to observe oral care and application of oral paste in another 'SDD-experienced' ICU.

A survey was used to determine expectations concerning and experience with SDD, and compliance to the study protocol. The survey to determine compliance to the study protocol was defined as the self-reported level at which nurses performed oral care according to the study protocol. Experience was focused on past and current experience with SDD. In the last week of each six-month study phase, all nurses and physicians working during a day (including night, day and evening shifts) received the questionnaire, which could be filled in anonymously [see Additional files 1 and 2]. With this single-day approach we expected to maximize response rates, because questionnaires could not be put aside but had to be returned the same day. In the second and third questionnaires (at the end of these study periods) it was also asked whether the nurse or physician had filled in a previous questionnaire. In the third questionnaire, nurses and physicians who participated in all three study periods were asked to grade workload, patient friendliness and effectiveness for SDD, SOD and standard care on a scale of 1 (low) to 10 (high). Patient friendliness was described as ease of application of oral hygiene and oral paste, and patient endurance of oral paste (taste, structure) to minimize additional stress in patients. Of note, nurses and physicians were not aware of the outcome results of the SDD-SOD trial at the time of the questionnaires.

### Questionnaire development

A comprehensive literature search in Medline and Cumulative Index to Nursing and Allied Health Literature was performed in August 2004. The following keywords were used: questionnaires [MeSH], attitude of health personnel [MeSH], intervention studies [MeSH], and SDD [free text]. The search did not reveal questionnaires on the attitudes of nurses and physicians towards a new intervention. Therefore, qualitative techniques were used to identify items, that is, problems encountered when executing the study protocol. The questionnaires were developed on observations of oral care and semi-structured interviews with seven nurses from four different hospitals at the start of the trial: four in a SDD-period, one in a SOD and two in a standard-care period. The observations revealed that nurses did not comply entirely with the oral hygiene protocol. During subsequent interviews the interviewer (IJ) pursued and clarified information on problems encountered during oral care and solutions to resolve reasons for non-compliance. Interviews were audio taped and transcribed verbatim. Transcripts were read and nurses' views regarding experience with SDD and problems met during oral care were identified and coded (by IJ and AS). Codes were continuously compared within and between transcripts. Agreement was reached between the researchers as to the major themes to be used in the questionnaires (concerning experience with and expectations of SDD), that is problems encountered during oral hygiene, non-compliance with the protocol, duration of oral care and expectations of SDD efficacy.

To maximize response rate, we designed a short questionnaire. For nurses, it contained four (standard care-period) to six (SDD and SOD-period) mostly closed questions, with a possibility to add comments in free text sections [see Additional file 1]. The nurses' questionnaire was pre-tested on three nurses (one research nurse and two ICU nurses), which resulted in a few linguistic changes only.

The questionnaires for physicians consisted of four closed and one open question in all study periods [see Additional file 2], addressing perceived clinical efficacy of SDD. Physicians were also asked to estimate ICU mortality rates in their standard care and SDD population, which were used to calculate the presumed relative reduction in mortality (PRRM), being the estimated mortality in SDD divided by the estimated mortality in standard care. The physicians' questionnaire was not pretested.

### Analysis

Data were analyzed using SPSS15.0 (SPSS Inc, Chicago, IL, USA).

Changes in opinion over time were analyzed by using chi-squared tests. Differences in time to perform oral hygiene and differences in grades were analyzed using medians (with interquartile ranges (IQR)) and non-parametric tests (Kruskal-Wallis tests, Friedman tests and Wilcoxon tests). A *P *value of less than 0.05 was considered statistically significant.

## Results

A total of 1,450 questionnaires were sent to nurses and 1,024 were returned (71%): 372 after period 1, 339 after period 2 and 313 after period 3. Of 307 questionnaires sent to physicians, 253 (82%) were returned: 85 after period 1, 89 after period 2 and 79 after period 3 (Table 2). About one-quarter (27% nurses, 24% physicians) of those who received the questionnaires completed them two or three times.

**Table 2:** Response and expectations of the effect of SDD per study period

	Nurses				Physicians				Total			
			
	1^st^	2^nd^	3^rd^	*P *value^⁪^	1^st^	2^nd^	3^rd^	*P *value^⁪^	1^st^	2^nd^	3^rd^	*P *value^⁪^
Response - no. (%)	372 (74)	339 (73)	313 (65)		85 (89)	89 (82)	79 (77)					

Prior experience SDD - %	53	74	87		68	85	90					

Effect SDD - no. (%)												
No effect	101 (33)	80 (26)	63 (22)	0.017	12 (14)	12 (14)	3 (4)	0.065	113 (29)	92 (23)	66 (18)	0.004
Decrease pneumonia	135 (43)	151 (49)	165 (58)	0.002	64 (75)	71 (80)	65 (84)	0.354	199 (50)	222 (56)	230 (68)	0.001
Increase resistance	68 (22)	68 (22)	48 (17)	0.209	14 (17)	19 (21)	21 (27)	0.247	82 (21)	87 (22)	69 (19)	0.624
Decrease resistance	24 (8)	21 (7)	25 (9)	0.672	11 (13)	13 (15)	14 (18)	0.640	35 (9)	34 (9)	39 (11)	0.524
Increase survival*	81 (26)	83 (27)	97 (34)	0.062	36 (42)	45 (51)	47 (61)	0.059				
Other	21 (7)	35 (11)	25 (9)	0.129	9 (11)	11 (12)	13 (17)	0.478	30 (8)	46 (12)	38 (11)	0.145

Median PRRM (IQR)					3.0 (0-25)	12.9 (0-25)	16.7 (0-28.5)	0.113				

IQR, interquartile range; PRRM, presumed relative reduction in mortality; SDD, selective decontamination of the digestive tract.

* Increase survival physicians based upon calculation PRRM.

^⁪ ^significance based upon chi-squared test (effect) or Kruskal-Wallis test (median PRRM).

### Expectations on SDD efficacy

The expected effect of SDD on patient outcome, as asked after every study period, increased during the study (*P *= 0.004; Table 2). The proportion of physicians that expected SDD to have no effects on clinical outcomes decreased from 14% after the first two periods to 4% at the end of study (*P *= 0.065). For nurses, these proportions were 33%, 26% and 22%, for periods 1, 2 and 3, respectively (*P *= 0.017). The most frequently reported expected effect of SDD was a reduction in the incidence of ventilator-associated pneumonia (VAP), and these proportions increased during the study (*P *= 0.001). Regarding improved ICU survival, both nurses and physicians tended to have increasing confidence in a positive effect of SDD on patient survival (*P *= 0.062 and *P *= 0.059, respectively). This corroborated the median calculated PRRM, as reported by physicians, which tended to increase from 3.0% (IQR 0 to 25) after period 1 to 16.7% (IQR 0 to 28.5) at the end of the study (*P *= 0.113).

The proportion of physicians that expected SDD to affect antibiotic resistance in their unit did not change significantly during the conduct of the trial. An increase in resistance was expected by 17% after period 1 and 27% at the end of study (*P *= 0.25) and a decrease in resistance was expected by 13% and 18% (*P *= 0.64) at these time points.

As we assumed that opinion on effect of SDD might be influenced by previous experience, we analyzed whether experience with SDD (either before or during the trial) was associated with expectations of SDD effects, which appeared not to be the case (chi-squared analysis, *P *= 0.74 and *P *= 0.98 for physicians and nurses respectively, data not shown). Trial results were not communicated, but neither intervention nor outcome were blinded for physicians and nurses. Data revealed that there was no correlation between the SDD-induced change in 28-day survival (observed effect in the trial) and the expected effect (by questionnaires) per hospital (*r *= 0.24, *P *= 0.43), nor between the observed effect and PRRM (*r *= -0.28, *P *= 0.36).

As additional effects of SDD, nurses mentioned better oral care, whereas physicians mostly mentioned a decrease in other infections (beside VAP), like urinary tract infections (Table 3).

**Table 3:** Free-text responses on additional effect of SDD - no

	Nurses	Physicians
No idea	82	1
Better oral hygiene	39	-
Increase colonization Enterococci/other bacteriae	3	6
Decrease other infections (besides VAP)	15	10
Other infection pattern	-	2
More frequent growth of yeasts	13	3
Less frequent growth of yeasts	2	-
Decrease length of stay	6	9
Increase length of stay	-	1
Better bacterial monitoring/antibiotics regimen	2	3
Increase diarrhea/change intestinal flora	3	-
Decrease multi organ failure	1	-
Decrease complications	-	1
Increase complications(wrong application)	-	1
Decrease morbidity	-	1
Decrease mechanical ventilation	-	2

SDD, selective decontamination of the digestive tract; VAP, ventilator-associated pneumonia.

### Self-reported compliance to protocol

Problems during oral care, as reported by nurses, occurred frequently. It was reported that in particular non-sedated patients experienced oral care as annoying (56%), disliked the flavor of the oral paste (46%) and/or suspension (22%), refused to cooperate during oral care (36%) or were nauseous (13%) (Table 4). Despite these problems, the self-reported adherence to the study protocol was 70%. Of nurses who did not comply, an average of 8% (7% in SDD, 8% in SOD) reported to have discontinued application and 6% (8% in SDD, 5% in SOD) reported to have modified the study protocol, by using a suspension instead of oral paste for oral care. The remaining 16% forgot to apply the oral paste on one occasion or at the right time point. Most modifications of the study protocol were made in non-intubated, non-sedated patients who refused the oral paste. These modifications did not seem to be influenced by expectations of nurses: the expected effect of SDD was not associated with being fully adherent to the study protocol (*P *= 0.65).

**Table 4:** Application of study protocol by nurses per intervention period

	SDD	SOD	Standard care	*P *value^⁪^
Extra time in minutes^‡ ^- median (IQR)	5.0 (2-5)	3.0 (0-5)	3.0 (0-5)	0.000

Problems^‡ ^- % of times reported	79	74		
- Patient disliked taste of oral paste - %	48	44		0.336
- Patient disliked suspension - %	22	--		--
- Patient was nauseous - %	17	9		0.003
- Patient found oral care annoying - %	54	58		0.318
- Patient did not cooperate with oral care - %	37	34		0.377

Change in application Orabase^‡ ^- %	31	29		0.305
- once not given - %	14	12		
- given at another time - %	2	4		
- discontinued - %	7	8		
- other - %	8	5		

IQR, interquartile range; SDD, selective decontamination of the digestive tract; SOD, selective oropharyngeal decontamination.

‡ Extra time, problems and change in application as reported by nurses.

^⁪ ^significance based upon chi-squared (problems, changes) or Kruskal-Wallis test (median extra time).

### Time needed for oral care

The estimated median time needed to perform oral care according to the protocol (which included applying oral paste every six hours during the SDD and SOD period) was 3.0 (IQR 0 to 5) minutes for both standard care and SOD and 5.0 (IQR 2 to 5) minutes for SDD (*P *< 0.001; Table 4). Estimated median additional times needed for oral care during SDD differed per center from 1.7 to 7.3 minutes. SDD was considered more time consuming than SOD and standard care in six centers and SOD was considered less time consuming than standard care in five.

### Grades for perceived workload and patient friendliness

Both physicians and nurses graded the estimated workload lowest for standard care and highest for SDD (Table 5). Although median differences in grades for SDD and SOD were small (5 and 4 for nurses and 5.5 and 5.0 for physicians, respectively), there was a tendency both in nurses and physicians to value workload during SDD higher as compared with SOD (*P *< 0.001 for nurses and *P *< 0.01 for physicians). Free text from nurses revealed that removing rests of oral paste from the oral cavity (before applying new paste) and increased prevalence of diarrhea contributed to a perceived higher workload during SDD. There was no relation between expected effect of SDD and the grade given for workload during SDD, neither in nurses nor in physicians.

**Table 5:** Median grades (interquartile ranges) for the three intervention periods

	N	SDD	SOD	Standard care	*P *value^⁪^
		median (IQR)	median (IQR)	median (IQR)	
**Nurses**					
Workload^a^	207	5.0 (4.0-7.0)	4.0 (3.0-6.0)	2.0 (1.0-4.0)	0.000
Pt friendliness^b^	197	4.0 (2.0-5.0)	4.0 (3.0-6.0)	7.0 (3.0-9.0)	0.000

**Physicians**					
Workload^a^	30	5.5 (3.8-7.0)	5.0 (3.0-6.0)	2.5 (2.0-4.0)	0.000
Pt friendliness^b^	27	6.0 (4.0-7.0)	6.0 (4.0-6.0)	8.0 (6.0-9.0)	0.003

IQR, interquartile range; N, number of responses; pt, patient; SDD, selective decontamination of the digestive tract; SOD, selective oropharyngeal decontamination.

^⁪ ^significance based upon Friedman test.

^a ^Workload measured on a scale from 1 (low) to 10 (high).

^b ^Patient friendliness measured on a scale from 1 (poor) to 10 (excellent).

SDD and SOD were considered significantly less patient friendly than standard care, both by nurses and physicians, with median values for SDD and SOD of 4 in nurses (IQR 2 to 5 and 3 to 6, respectively) and 6 in physicians (IQR 4-7 and 4-6, respectively) and for standard care of 7 in nurses (IQR 3 to 9) and 8 in physicians (IQR 6 to 9). There was a difference in grade for patient friendliness given by nurses for SDD as compared with SOD (Wilcoxon test, *P *< 0.001), whereas for physicians there was no difference between the intervention periods. In free text, nurses often mentioned the taste and color of the oral paste as patient unfriendly, especially in non-ventilated and non-sedated patients. Furthermore, the suspension of SDD was considered unfriendly, especially when the nasogastric tube was removed and the patient was asked to swallow the suspension.

## Discussion

The results of our study reveal that physicians and nurses considered SDD to have a higher workload and to be less patient friendly than standard care. Moreover, expectations on the effects of SDD, especially on pneumonia, changed during the study, both among physicians and nurses, independent of study order and without knowledge of trial results.

Nurses associated SOD with a lower increase of their workload than SDD. The (statistically significant) difference in perceived duration of oral care in the SDD and SOD period is remarkable, because the oral care protocol did not differ in both interventions. An explanation may be that nurses included intuitively the time needed for the preparation and administration of the gastric solution and intravenous antibiotics.

Previous studies have reported nurses' perception of oral care practices as being difficult and unpleasant to perform [17-19]. This was confirmed in our survey, with nurses believing that oral care, especially application of oral paste, was unpleasant and 'unfriendly' for patients. Although oral hygiene was the same in SDD and SOD, the perception of patient friendliness differed. These results suggest that introduction of SDD and SOD should be accompanied by education in which the importance of oral care is emphasized in order to reduce the perception that oral care is unpleasant [20].

Thirty percent of the nurses reported a protocol violation in the application of oropharyngeal decontamination. Nurses mostly mentioned that they failed to administer the oropharyngeal paste only once. More obvious non-adherence appeared to be associated with the sedation level and ventilation status of a patient: the self-reported discontinued application of the oropharyngeal paste occurred predominantly in non-ventilated and non-sedated, alert patients. Based on notifications on the patient record forms during the trial, we estimated that oropharyngeal decontamination had not been administered in 2.5% and 4.3% of all patient days during SDD and SOD, respectively [16]. Given these figures and the additional comments that non-compliance mainly occurred in non-ventilated, non-sedated patients, it is unlikely that these incidental failures to apply medication affected the effectiveness of the interventions.

At the start of the trial, already most nurses and physicians expected SDD to effect patient outcome and this group had a relative increase of 15% towards the end of the trial. The median PRRM tended to increase during the conduct of the trial, and came close to the 13% relative risk reduction in 28-day mortality as determined in the trial [16]. As physicians were asked to estimate this benefit after each study period, we assume that the increasing proportion of physicians that had had experience with SDD explains this gradual change.

An important objection against the widespread use of SDD or SOD has been the possibility of an increase of antibiotic resistance. This was an important reason for physicians in the UK for not using SDD [21]. Our survey revealed non-conclusive results on the physicians' expectations on the effects of SDD on antibiotic resistance. During the study increasing proportions of physicians expected that SDD would be associated with either an increase or a decrease of antibiotic resistance. Yet, the actual observed effects revealed that carriage levels with antibiotic-resistant pathogens in the intestines and the respiratory tract reduced during SDD and SOD [16].

Strengths of our study include the high response rates for both nurses and physicians and the fact that this is, up until now, the only prospective evaluation of perceived opinions related to SDD and SOD. There are several limitations to our study. First, it was not possible to fully validate the questionnaires. No (multi-item) factor analysis was performed on the items of the questionnaire, because only one question per topic was included. On the other hand, to enhance validity, we used triangulation: a combination of, in our study, two methods (observations and subsequent interviews) to develop consistent and comprehensive questionnaires about problems and expectations [22,23]. Furthermore, the questionnaire for physicians was not pretested, unlike the questionnaire for nurses.

A second limitation is the variability in respondents, because after every study period nurses and physicians working on a selected day were invited to fill in the questionnaire. Therefore, different nurses and physicians might have filled in the first, second and third questionnaires and changes in expectations might be influenced by the different respondents. However, because of the high response rate in all participating hospitals during each of the study periods, it is unlikely that important bias has been introduced. In addition, restricting the analysis to professionals who filled in the questionnaire two or even three times revealed similar conclusions (data not shown).

## Conclusions

Among multiple different interventions aiming to reduce the incidence of VAP in ICU patients, SDD and SOD are currently the only two associated with demonstrated improvements in patient survival. Yet, widespread and correct implementation of these interventions will critically depend on the acceptance by health care workers that need to perform these procedures. Therefore, we recommend education about the importance of oral care and to provide clear information about the effects of SDD and SOD on patient outcomes.

## Key messages

• Nurses considered SDD to result in a higher workload and to be less patient friendly as compared with SOD and standard care.

• Physicians considered both SDD and SOD to result in a higher workload and be less patient friendly as compared with standard care.

• The expectations of both nurses and physicians on the effects of SDD on patient outcome, especially on pneumonia and patient survival, changed over time.

• Confidence of nurses and physicians in effects of SDD increased over time.

## Abbreviations

IQR: interquartile range; PRRM: presumed relative reduction in mortality; SDD: selective decontamination of the digestive tract; SOD: selective oropharyngeal decontamination; VAP: ventilator-associated pneumonia.

## Competing interests

The authors declare that they have no competing interests.

## Authors' contributions

IJ participated in the design and coordination of the study, performed the statistical analysis and drafted the manuscript. AS participated in the design of the study, helped to do statistical analysis and to draft the manuscript. JK conceived of the study, participated in its design and helped to draft the manuscript. LV, PD, RW, MPB, RS, DBH, NM, JV, KK, MI, GK, TW, HH and AB all participated in the coordination of the trial and helped to draft the manuscript. PP participated in the coordination of the trial and in the statistical analysis and helped to draft the manuscript. MJB conceived of the study, participated in its design and in statistical analysis and helped to draft the manuscript. All authors read and approved the final manuscript.

## Supplementary Material

Additional file 1 **Nurses' questionnaire**. Questions sent to nurses after each study period (translation of original Dutch questionnaire). Click here for file

Additional file 2 **Physicians' questionnaire**. Questions sent to physicians after each study period (translation of original Dutch questionnaire). Click here for file
