# Supplementary material for: Physicians' and nurses' opinions on selective decontamination of the digestive tract and selective oropharyngeal decontamination: a survey
Source: Crit Care. 2010 Jul 13;14(4):R132. doi: 10.1186/cc9180 (PMC2945100; doi:10.1186/cc9180)
Supplement: Additional file 1 — Nurses' questionnaire. Questions sent to nurses after each study period (translation of original Dutch questionnaire). [file cc9180-S1.doc]

**Nurses’ Questionnaire**

Hospital:

Study period: …………………………

Date: ……………………………………

*(translation; original questionnaire in dutch)*

1. *(Question in 2^nd^ and 3^rd^ study period)* Did you complete this questionnaire previously after a prior study period?

❑ Yes ❑ No

2. Did you previously (before this trial) apply SDD?

❑ no

❑ yes, what was your experience with SDD at that time?

❑ good, because

❑ neutral, because

❑ not good, because

3. *(Question in SDD and SOD period)* Keep in mind the last patient you cared for and who was included in the SDD/SOD-trial. Was the following applicable for this patient:

- patient disliked the flavour of the oral paste (Orabase) yes / no
- patient disliked suspension yes / no
- patient was nauseous yes / no
- patient found oral care annoying yes / no
- patient did not cooperate with oral care yes / no

4. *(Question in SDD and SOD period)* When at least one of the questions in 3 is answered with “yes”: was this a reason to change application of oral paste (Orabase) or suspension?

❑ not applicable (all questions in 3 answered with “no”)

❑ no, oral paste and suspension were applied according to protocol

❑ yes, application was changed, namely:

❑ oral paste / suspension was not applied once

❑ oral paste / suspension was applied at another moment

❑ other, namely

5. How many minutes do you need extra at a time to perform oral care due to the SDD/SOD-trial?

❑ no time extra

❑ about minutes extra per time

6. What do you expect of the effectiveness of SDD?

❑ no effect

❑ indeed effect, namely (more answers possible)

❑ decrease in pneumonia

❑ increase in antibiotic resistance

❑ decrease in antibiotic resistance

❑ increase of survival of patients

❑ other, namely

7. *(Question in 3^rd^ study period)* Did you participate in all three study periods of the SDD/SOD-trial?

❑ no, not applicable

❑ if yes, can you give a grade for each of the study periods for the following aspects?

  SDD-period SOD-period Standard Care

Workload (1=small, 10=high workload) ……….. ………… ……………

Patient friendliness (1=poor, 10=excellent) ……….. ………… ……………

Effectiveness (1=poor, 10=excellent) ……….. ………… ……………

8. Do you have other information you like to add concerning the SDD/SOD-trial?
