# Supplementary material for: Physicians' and nurses' opinions on selective decontamination of the digestive tract and selective oropharyngeal decontamination: a survey
Source: Crit Care. 2010 Jul 13;14(4):R132. doi: 10.1186/cc9180 (PMC2945100; doi:10.1186/cc9180)
Supplement: Additional file 2 — Physicians' questionnaire. Questions sent to physicians after each study period (translation of original Dutch questionnaire). [file cc9180-S2.doc]

# Physicians’ Questionnaire

Hospital:

Study period: …………………………

Date: ……………………………………

*(translation; original questionnare in dutch)*

1. *(Question in 2^nd^ and 3^rd^ study period)* Did you complete this questionnaire previously after a prior study period?

❑ Yes ❑ No

1. What is your profession in ICU?

❑ intensivist

❑ specialist not intensivist

❑ Resident

❑ Intern

3. Have you previously worked with SDD?

❑ yes, during this trial

❑ yes, in this unit before the trial

❑ yes, elsewhere

❑ no, no prior experience with SDD

1. How do you estimate current ICU mortality of the included patient group?

About %

How do you estimate ICU mortality in this patient group after application of SDD?

About %

5. What do you expect of the effectiveness of SDD?

❑ no effect

❑ indeed effect, namely (more answers possible)

❑ decrease in pneumonia

❑ increase in antibiotic resistance

❑ decrease in antibiotic resistance

❑ other, namely

6. Where do you base your expectation of effectiveness and mortality upon (more answers possible):

❑ published trials

❑ own experience

❑ experience of others

❑ other, namely

7. *(Question added in 3^rd^ study period)* Did you participate in all three study periods of the SDD/SOD-trial?

❑ no, not applicable

❑ if yes, can you give a grade for each of the study periods for the following aspects?

  SDD-period SOD-period Standard Care

Workload (1=small, 10=high workload) ……….. ………… ……………

Patient friendliness (1=poor, 10=excellent) ……….. ………… ……………

Effectiveness (1=poor, 10=excellent) ………… ………… ……………

1. Do you have other information you like to add concerning the SDD/SOD-trial?
